# Opportunities and challenges of integrating genetics education about human diversity into public health nurses’ responsibilities in Japan

**DOI:** 10.1186/s12912-019-0391-6

**Published:** 2019-12-09

**Authors:** Hiromi Goda, Hiromi Kawasaki, Yuko Masuoka, Natsu Kohama, Md Moshiur Rahman

**Affiliations:** Graduate School of Biomedical and Health Sciences, Hiroshima University, 1-2-3, Kasumi, Minami-ku, Hiroshima, 734-8553 Japan

**Keywords:** Genetics, Nursing, Genetic counseling, Public health nurses, Qualitative research

## Abstract

**Background:**

Many genetic tests are now available due to evolution by the Human Genome Project. However, the growing use of genetic testing and screening may not only lead to improvements in public health and health promotion, but also trigger grave ethical, legal, and societal concerns. The involvement of community healthcare providers is expected in the future because they have direct contact with the residents through their health programs. The aims of the current study were to clarify how public health nurses perceive the relationship between genetics and professional duties and to identify opportunities and challenges of integrating genetics education into their professional duties.

**Methods:**

In 2017, data were collected through a focus group interview. Content related to the ‘presence or absence of health consultation related to genetic issues as part of actual job duties’ and ‘training for handling genetic-related health consultations’ was extracted. Entire sentences were coded and categorized based on similar semantic content.

**Results:**

Public health nurses had difficulties in recognizing genetic issues in public health activities. In some cases, genetic contents were included in conversation and consultation with the general public. Through the professional education and experiences, public health nurses needed specialized education, post-graduation studies and mentoring about genetics.

**Conclusions:**

Public health nurses whose professional duties do not directly relate to genetics were exposed to genetics-related episodes on a regular basis without their awareness. The provision of accurate information and knowledge related to genetics by public health nurses would also provide an opportunity for clients to be informed about their latent genetic risks. Hence, there is a need for practical resources, the establishment of collaboration networks, and the development of professional organizations for public health nurses.

## Background

There are many human genome projects develop tools for researchers to identify new strategic paths for the diagnosis, treatment, and prevention of human diseases and to understand their genetic causes. As a result, there are now more than 60,000 genetic tests are available and allowing the detection of more than 18,000 genes suspected triggering genetic diseases [1]. The increasing number of genetic tests offers companies direct access to consumers (DTCs), providing affordable and easy-to-use genetic testing, without the guidance of healthcare professionals. DTC tests provide predictive testing to determine the predisposition to single-gene disorders such as cystic fibrosis and multifactorial diseases, for example, depression and cardiovascular disease [2]. The International HapMap Project ran concurrently with the Human Genome Project. This project covered the diagnosis of diseases, discovered of genes responsible to diseases, involved in the protection against illnesses, associated with drug response and dramatically accelerated the development of new preventive and therapeutic strategies [3–5].

To study the genes’ role in diseases, Mayo Clinic is scanning 20,000 genes among thousands of patients, and only 59 genes’ findings will be shared. Mayo will examine certain disease-causing gene variants for heart disease or breast cancer, and share the results to the patient. The use of genetic testing for screening has increased public health contributions [6].

The growing use of genetic testing and screening may not only lead to improvements in public health and health promotion, but also trigger grave ethical, legal, and societal concerns [7–9]. In 1997, the World Health Organization (WHO) proposed the establishment of ethical criteria at the national and international levels to verify the ethical and social benefits of advancements in medical genetics [10]. To address these emerging ethical issues, many countries have offered solutions for the handling of genetic information [11, 12]. For instance, in Europe, the following three solutions are typically offered: prohibition of any use of genetic information by insurers’ outright, legislation prohibiting genetic information below a certain amount of coverage and Moratoria. The United Nations Educational, Scientific and Cultural Organization (UNESCO) adopted the Universal Declaration on the Human Genome and Human Rights (UNESCO, 1997) to recognize the importance in managing genetic information [13]. Other than laws, conventions, and declarations, improvements in individual ‘genetic literacy’ and the understanding of human genetic diversity could resolve the ethical issues associated with genetic and medical technologies. In addition, ‘Genetics education for the public can best be achieved through education in schools’ [14]. In the future, genetic education will be needed to improve genetic literacy not only for healthcare providers and patients, but also for the general people [15]. WHO highlights the importance of the future role of community healthcare providers as they have direct contact with the residence through their health programs [14].

In Japan, the roles and responsibilities of public health nurses are legally allocated by their affiliated local municipalities. The established system allows public health nurses a speedy and accurate response to health risks, including disasters. In collaboration with stakeholders, public health nurses affiliated with local municipalities’ plan, provide, and evaluate public health services for promoting health. These actions address the common health issues experienced by the general people [16]. However, public health nurses do not have enough knowledge on genetics and sufficient time to address the genetics services demand by the community [17]. Public health nurses belonging to prefectural authorities provide a wide range of specialized public health services as part of their daily work, including measures to counter intractable diseases, tuberculosis, infectious diseases, acquired immune deficiency syndrome (AIDS), etc. [16]. So, they have a lot of opportunities to obtain knowledge on genetics. In contrary, external to their professional duties, the public health nurses who are working as community health providers are facing challenges during health promotion activities specially on genetics-related diseases [17]. So, this study aimed to clarify how public health nurses affiliated with local municipalities perceive the relationship between genetics and professional duties and to identify opportunities and challenges of integrating genetics education into the public health nurses’ professional duties.

## Methods

### Design

This present study employed a qualitative descriptive approach for data collection and analysis in order to clarify any opportunities or challenges related to integration of genetics education for the public health nurses outside of their current work.

### Participants

Public health nurses are in charge of maternal and child health, elderly health, and mental health and usually experienced all kinds of assignments by rotation duties. Public health nurses who were working at local municipalities in Kagoshima Prefecture, Japan, were recruited into the study through the purposeful sampling method in 2017. Six public health nurses were included in the study who provided informed consent. The participants were selected on condition that they were not to be involved in duties related to genetics and not in management positions. As approval to participate in the present study was obtained from public health nurses working at local municipalities in Kagoshima Prefecture, only participants who provided informed consent were recruited. During the interview, they were named and anonymity was guaranteed by using the initial letters of their positions followed by the Arabic numerals from “phn1” to “phn6”.

### Data collection

Data were collected from a focus group interview (FGI). A moderator led the FGI involving all participants based on an interview guide (Additional file 1). The FGI lasted approximately 30 min, and the location of the interview was chosen to ensure privacy. A voice recorder was used to record the FGI after obtaining the permission of all participants. During the FGI, the participants’ anonymity was assured by using number cards instead of names.

The content of the FGI focused on the following: 1) experience in counseling or providing support related to genetics; 2) impressions of counseling related to genetics; 3) awareness that counseling and support are linked with genetics; 4) aspects of genetic-related counseling and support that the participants found challenging; 5) ideal timing and content of genetic education; and 6) genetic knowledge required in genetic education.

### Data analysis

An accurate transcript was produced from the recordings, and qualitative content analysis was conducted based on the transcript. The analysis was conducted as follows. Content related to the ‘presence or absence of health consultation related to genetic issues as part of actual job duties’ and ‘training for handling genetic-related health consultations’ was extracted. Entire sentences were coded and categorized based on similar semantic content.

### Rigor

The validity of the data interpretation and the consistency of the analyzed results were tested repeatedly by multiple investigators. The present study sought to establish content reliability and validity. Based on the verbatim records, we extracted all the phrases including topics that indicate genetic problems and cognition, read the meaning from the context before and after, and examined the meaning and coded it. In addition, codes were collected according to the similarity of semantic content, and subcategories were extracted, and then categorized. In order to ensure reliability and validity, a total of three people examined each coded sentence. One of them is specialized in genetic nursing and qualitative research, and other two are specialized in public health nursing.

### Ethical considerations

The approval was obtained from the Hiroshima University Ethics Committee for Epidemiological Research prior to the commencement of the study (No. E-800-1). The participants were given a verbal explanation in advance and assured that the content of the study would not be used for any other purpose. A written explanation was also provided to the participants, and data were collected upon receipt of their consent. Written informed consent was obtained from all the participants.

## Results

### Participants

Six public health nurses (five women [83.3%], one man [16.7%]; mean years of experience: 14.7 ± 9.1 years) participated in the study, three (50.0%) of them had previous experience working as nurses in hospitals. The FGI lasted 38 min.

### Extraction of categories

The analysis of the results revealed four categories consisting of 50 codes, and 10 subcategories (Table 1). In the manuscript, the categories are enclosed in (brackets), the subcategories in [square brackets], and the direct quotations in *“quotation marks”*.

**Table 1:** Current situations and problems of health consultation on genetics in public health activities

Category	Subcategory
I. Difficulty in recognizing genetic issues in public health activities	1. Not aware of the causes of the diseases
	2. No awareness of genetics while performing regular duties
II. Genetic knowledge will be useful in health promotion if specific support services are available	1. The existence of genetics as a topic of consultation in everyday interactions
	2. The consultation question may have had genetic aspects
	3. There were aspects of genetics that they knew because they had an understanding of families and the community
III. Varying levels of expectations in genetic education	1. Genetic education should be incorporated as part of the mandatory education system because it teaches diversity
	2. Genetic education is needed as part of basic training for public health nurses
IV. A desire to obtain support-related information when needed	1. Public health nurses need genetic education as part of their post-graduation studies
	2. Genetic knowledge is not frequently used in the workplace
	3. Need for mentoring where one could ask questions when confronted with actual challenging situations

Category I: (Difficulty in recognizing genetic issues in public health activities)

- *“I may not have consciously paid much attention to serious consultation questions from the general public related to genetics, due in part to not carefully considering that questions were related to genetics* […]*”* (phn4).
- *“*[…] *When providing health consultations, I am not very aware of whether a question is related to genetics”* (phn6).

Based on the above, the subcategory was [Not aware of the causes of the diseases].

Furthermore,

- *“I do not know any details regarding genetic diseases* […]*”* (phn2).
- *“*[…] *I do not have specific images when I hear the word ‘genetics’”* (phn2).
- *“I do not have an image of what genetics is. The reason is because no one in the general public has ever used the word ‘genetics’ in real practice while I provide consultations as a public health nurse”* (phn3).
- *“I cannot get an exact mental picture of what a genetic disease looks like* […]*”* (phn4).
- *“*[…] *I have taken family histories related to diabetes from the patient’s family; however, during that process, I was not consciously thinking of genetics”* (phn4). Based on the above, the subcategory was [No awareness of genetics while performing regular duties].

Category II: (Genetic knowledge will be useful in health promotion if specific support services are available).

Contrary to Category I,

- *“*[…] *During a baby health examination, I heard a mother talk about her concerns regarding genetic diseases of babies as she had a late-age childbirth* […]*”* (phn5).
- *“*[…] *When I was working in the mental health field, a member of the general public once asked me about a genetic issue. However, it was not a specific question* […]*”* (phn5).

As noted above, the subcategory [The existence of genetics as a topic of consultation in everyday interactions] emerged during conversations with the general people.

- *“*[…] *I used to be asked questions on cancer and diabetes on a regular basis* […]*”* (phn4).
- *“I used to think that high blood pressure was ‘familial’ rather than genetic. However, after careful reflection, it may be a genetic issue”* (phn3).
- *“*[…] *I remember I was once asked whether a person was likely to die from stomach cancer as the person’s father died of stomach cancer* […]*”* (phn4).
- *“*[…] *I did not think of anything when I handed out the check-up results. However, later I noticed that the platelet count was low, similar to the person’s mother and sister. I thought that this might be a genetic issue”* (phn2).
- *“During history-taking, I used to be asked whether the person was likely to suffer the same disease as a family member (for instance, a father’s high blood pressure and a sister’s breast cancer). At that time, I realized this was a genetic question* […]*”* (phn5). Upon careful consideration, the subcategory was [The consultation question may have had genetic aspects].

The public health nurses had not been directly involved in health consultations related to genetics. However,

- *“*[…] *There were many people with retinal pigmentary degeneration, an intractable disease, living in the same village* […]*”* (phn5).
- *“*[…] *When a family tree was traced back in detail, I found that there were siblings with autism or developmental disorders and parents with developmental disorders* […]*”* (phn2). Some public health nurses mentioned that [There were aspects of genetics that they knew because they had an understanding of families and the community] through their work.

Category III: Varying levels of expectations in genetic education

- *“Education for genetic diseases should be incorporated into the education system from elementary school through high school if learning is expected to include human rights and race issues* […]*”* (phn4).
- *“*[…] *Similar to sex education, children should learn about genetic diseases from the time they start elementary school because they are able to accept differences in race and human rights as their minds are flexible and free of prejudice* […]*”* (phn4). Therefore, [Genetic education should be incorporated as part of the mandatory education system because it teaches diversity]. Furthermore,
- *“Basic knowledge of genetic diseases is part of specialized education for public health nurses* […]*”* (phn3).
- *“Public health nurses should receive specialized training at educational institutions for public health nurses and nursing schools so that they can use their expert knowledge when asked about genetic issues* […]*”* (phn1).
- *“*[…] *Students should study the nature of and prevention methods for genetic diseases as part of their public health education. This should be included in specialist training system and curriculum* […]*”* (phn4). Regarding specialized genetic knowledge, public health nurses believe it [Genetic education is needed as part of basic training for public health nurses].

Category IV: (A desire to obtain support-related information when needed)

- *“One needs to be equipped with knowledge on what to do after identifying that a member of the general public has a genetic disease* […]*”* (phn4).
- *“*[…] *More studying is needed so that better support, such as making referrals to relevant institutions, can be provided* […]*”* (phn5).
- *“*[…] *Studying should not be limited to one’s time in school because the latest medical information becomes available even after graduation. It is also necessary to listen carefully to the voices of the general public during consultations. Public health nurses need to acquire new information at times”* (phn4). In addition to specialized education, [Public health nurses need genetic education as part of their post-graduation studies].
- *“When prenatal diagnostic tests became available, there was a manual that outlined ways to respond to questions related to the prenatal tests* […]*”* (phn5).
- *“*[…] *As they include very technical details, public health nurses can acquire various insights by referring to the manuals* […]*”* (phn5).
- *“*[…] *Not many genetics-related questions are asked on a daily basis. Therefore, it would be very useful to have a collection of case studies with examples on how similar cases were handled and resolved”* (phn6) as based on the above, the subcategory was [Genetic knowledge is not frequently used in the workplace].
- *“*[…] *Given the wide scope and challenging nature of the study, it would be ideal to establish a place that offers genetic knowledge”* (phn3).
- *“*[…] *A place is needed where public health nurses, regardless of their years of experience, can access genetics information on a regular basis and ask highly technical questions”* (phn6). In other words, there is a [Need for mentoring where one could ask questions when confronted with actual challenging situations].

## Discussion

The study intended to elucidate how the local municipality affiliated public health nurses perceive the relationship between genetics and professional duties, and to explore the related challenges and opportunities. The study findings revealed that public health nurses encountered difficulties in recognizing genetic issues while providing their professional services. They mentioned that they needed to pursue specialized education and mentoring to handle their clients properly in some cases. During health consultations, public health nurses were more concerned about how to connect patients with supporting services than focusing on the causes of the diseases. Public health nurses may not have been thinking about the causes of the diseases (Subcategory I-1) since the causes and treatments are often managed by hospital staffs. Public health nurses mainly provide lifestyle-related supports to patients. Moreover, public health nurses expressed that they were not conscious of genetic issues when performing their regular duties (Subcategory I-2). This could be attributed to the following two reasons. First, public health nurses are involved with patients through services that provide lifestyle support for recovery. Second, public health nurses provide individualized support depending on the severity and type of genetic disease. Genetic diseases, which require more individualized support, may not focus during public health activities that characteristically possess a population approach. Considering the different duties of public health nurses, it became apparent that they are faced with situations that make it harder for them to be aware of genetic issues while performing their duties (Category I).

Public health nurses working in the field of mother-and-child healthcare and mental health were involved with genetic issues even though not only health consultations, but also conversations with general people during medical checkups and health educations. This is probably because talking about concerns and stress during medical checkups, and health workshops create opportunities to strike up conversations with public health nurses. Cancer, diabetes, and hypertension are diseases well-known to general people. This is probably because people consult with public health nurses about the results of their medical checkups or cancer tests. Consultation issues related to genetics emerged as part of regular dealings with patients (Subcategory II-1). There may have been consultations related to genetics if one paid closer attention to regular dealings with patients (Subcategory II-2).

Public health nurses perceived genetic diseases, not as ‘genetics’, but more to do with general people and family members living in the same community ‘having similar constitutions’ (Subcategory II-3). Studies have shown that cardio-metabolic risks as obesity and high blood pressure differ between communities [18]. Correlation between parent and adolescent body mass index (BMI) is due to not only genetic condition, but also unhealthy diet and lifestyle have been demonstrated [19]. As people living in the same community share the same geographical features, climate, and traditions, it is possible that their constitutions and diseases are as well similar; an even stronger influence is observed within families. During health consultations related to genetics, public health nurses perceived the residents’ symptoms, and diseases were not limited to “genetic”. Public health nurses are able to understand these situations because they adopt a broad comprehensive view of the targets of their nursing practice such as families and communities.

In Turkey, public health nurses are involved in genetic counseling because there are no genetic counselors or genetic nurses [20]. At the same time, public health nurses in Japan were in charge of family planning such as determining the number and spacing of one’s children through birth control, genetic consultation, and training [21]. Ever since hospitals began conducting genetic diagnoses, public health nurses have not been involved because all duties associated with ‘genetic counseling’ were deemed consultations related to ‘gene diagnosis’. Because of the widespread availability of genetic testing, public health nurses may become involved in primary genetics counseling prior to genetic testing, delivery of genetic information, and consultation to enhance health promotion (Category II). This is in support of a widespread view of genetic diseases.

Originally, the roles of public health nurses are included advocacy and educating the community [22, 23]. The role of public health nurses in promoting human genetic diversity is very important. With the team approach adopted in genetic services, there are high expectations for community healthcare providers, including public health nurses [14]. The public health nurses will be able to provide basic knowledge and information on human genetics to their clients regarding their underlying genetic concerns.

Public health nurses noted that there are various levels in genetic education. For instance, the knowledge should be learned as a part of the mandatory nursing education system and to be acquired at a specialized level (Category III). Human genetic diversity should be learned from a young age, when minds are flexible and free of prejudice, not after children have grown into adults (Subcategory III-1). Studies have shown that many people in the community lack of more ethical issues compared with scientific knowledge [24]. In addition, lower level of genetic knowledge is related to older age and lower education level [24, 25]. The consequences of misconceptions of the general people are based on (i) a lack of formal scientific education, and (ii) the poor coverage of genetics in the media and on the Internet [23]. This opinion was shared by many other public health nurses. All children have an equal level of education. With the minimal influence of the media and Internet, children can learn free of prejudice. When children learn accurate information and pass it to adults, misunderstandings of genetic information in adults may decrease [26]. It is very important to learn from a young age, not only scientific knowledge, but also genetic education that includes ethical issues.

In the future, there will be a growing need for healthcare providers and specialist who have roles in interpreting genomic information for the general public as well as disseminating and promoting accurate information [27, 28]. Public health nurses need to acquire specialized genetic education through not only mandatory education, but also basic training, in order to become a public health nurse (Subcategory III-2). The American Nurses Association and the International Society of Nurses in Genetics have defined the need for nurses to acquire genetic knowledge as: “Nurses require genetics and genomics knowledge to identify, refer, support, and care for persons affected by, or at risk for, manifesting or transmitting conditions or diseases with a genetic component” [29].

Various other countries such as Taiwan, Italy, and Turkey acknowledged the need to incorporate genetics in nursing education [20, 30]. However, genetic education levels in nursing were found to be low because of deficiencies in nursing curricula and the lack of course content in nursing schools [31]. While public health nurses understood the need for genetic knowledge pertaining to nursing, they were also aware of the reality that the education system is not fully in place. On top of their specialized training as students, public health nurses felt the need to acquire more constructive genetic knowledge to assist them in practical connections with support services as part of their nursing duties (Subcategory IV-1). On the other hand, as genetic knowledge is rarely utilized in actual practice (Subcategory IV-2), it will be very difficult to spend sufficient time studying after graduation. Internet-based genetic education programs for various targets, such as nurses, university faculty members, and nursing students, are offered as resources from many organizations in the US, Australia, the UK, and other countries around the world [32]. By using these online resources, public health nurses can always acquire the latest information. Using globally available online resources as a foundation, there is also a need in Japan to develop resources that serve to the practical duties of public health nurses. As genetic knowledge is rarely utilized in the actual practice of public health nurses, the idea was raised to develop ‘resources that one could use as references or models when needed, such as case study collections or manuals’. The challenge will be to prepare easily accessible resources related to rare diseases that could be used together with general people. There is an expectation for not only resources, but also the availability of advisors that could ‘help answer questions when confronted with actual difficult cases’ (Subcategory IV-3). This issue could be resolved by cooperating with genetic counselors or certified nurse specialists currently handling ‘genetic counseling’. Public health nurses could work at comfort if all countries had specialized organizations such as the UK’s Genetics/Genomics Competency Centre (G2C2) and Genomics Medicine Centres (GMCs).

The results of the present study were based on the work experiences of the study participants. As the study targeted a limited number of participants in one municipality, there is a need to continue the further investigation by combining the present findings with quantitative research in different communities without biases.

## Conclusions

Public health nurses whose professional duties do not directly relate to genetics were exposed to genetics-related episodes on a regular basis without their awareness. The sharing of precise facts and understanding regarding human genetics by public health nurses would also contribute an opening for clients to be communicated concerning their hidden genetic issues. As a result, there is a need for practical resources, the establishment of collaboration networks, and the development of professional organizations for public health nurses to identify potential risks.

## Supplementary information

**Additional file 1.** Interview guide developed for this study

## Data Availability

Due to protect and maintain participants’ anonymity and confidentiality the data sets analyzed during the current study will not be shared. Data will only be available on a reasonable request from the corresponding author.

## Abbreviations

AIDS: Acquired immuno deficiency syndrome
BMI: Body mass index
DTC: Direct access to consumers
FGI: Focus group interview
G2C2: Genetics/Genomics competency centre
GMC: Genomics medicine centre
UNESCO: United Nations Educational, Scientific and Cultural Organization
WHO: World Health Organization

## References

[1] Genetic Testing Registry [Home page on the Internet]. Retrieved from: https://www.ncbi.nlm.nih.gov/gtr/. Accessed 3 Oct 2019.

[2] Hudson K, Javitt G, Burke W, Byers P (2007). ASHG social issues committee. ASHG statement on direct-to-consumer genetic testing in the United States. Am J Hum Genet.

[3] National Human Genome Research Institute (2012). Fact sheet - about the international hapmap project.

[4] Manolio TA, Brooks LD, Collins FS (2008). A hapmap harvest of insights into the genetics of common disease. J Clin Invest.

[5] Bailey JN, Pericak-Vance MA, Haines JL (2014). The impact of the human genome project on complex disease. Genes.

[6] Evans M, Mathews AW (2019). Doctors limit what to tell patients about their DNA test. Should they? The wall street Journal.

[7] American Medical Association (2013). Genetic discrimination.

[8] Goldsmith L, Jackson L, O'Connor A, Skirton H (2012). Direct-to-consumer genomic testing: systematic review of the literature on user perspectives. Eur J Hum Genet.

[9] Pascal S (2013). Direct-to-consumer genetic testing: a comprehensive view. Yale J Biol Med.

[10] World Health Organization (WHO) (1997). Proposed international guidelines on ethical issues in medical genetics and genetic services.

[11] Joly Y, Braker M, Le Huynh M (2010). Genetic discrimination in private insurance: global perspectives. New Genet Soc.

[12] Joly Y, Feze IN, Song L, Knoppers BM (2017). Comparative approaches to genetic discrimination: chasing shadows?. Trends Genet.

[13] United Nations Educational, Scientific and Cultural Organization (UNESCO) (2000). The universal declaration on the human genome and human rights: from theory to practice.

[14] World Health Organization (WHO) (2003). Review of ethical issues in medical genetics.

[15] Syurina EV, Brankovic I, Probst-Hensch N, Brand A (2011). Genome-based health literacy: a new challenge for public health genomics. Public Health Genom.

[16] Health, Labour and Welfare Statistics Association (2014). Trends in the health of the nation in Japan: Journal of health and welfare statistics extra edition.

[17] Sasaki N, Morifuji K, Tsubota S, Matsumoto T, Miyahara H (2015). Experiences of public health nurses providing genetic counseling in underpopulated areas of Nagasaki prefecture. J Japanese Soc Genet Nurs.

[18] Daniel CR, Prabhakaran D, Kapur K, Graubard BI, Devasenapathy N, Ramakrishnan L (2011). A cross-sectional investigation of regionalpatterns of diet and cardio-metabolic risk in India. Nutr J.

[19] Kosti RI, Panagiotakos DB, Tountas Y, Mihas CC, Alevizos A, Mariolis T (2008). Parental body mass index in association with the prevalence of overweight/obesity among adolescents in Greece; dietary and lifestyle habits in the context of the family environment: the Vyronas study. Appetite.

[20] Seven M, Akyüz A, Elbüken B, Skirton H, Öztürk H (2015). Nurses' knowledge and educational needs regarding genetics. Nurse Educ Today.

[21] Hokama T, Inoue Y, Takenaka S, Hirayama K, Osiro H, Maeuezato K (1982). Genetic counseling at the public health center. Ryukyu Univ J Health Sci Med.

[22] Ivanov LL, Oden TL (2013). Public health nursing, ethics and human rights. Public Health Nurs.

[23] Dougherty MJ, Lontok KS, Donigan K, McInerney JD (2014). The critical challenge of educating the public about genetics. Curr Genet Med Rep.

[24] Haga SB, Barry WT, Mills R, Ginsburg GS, Svetkey L, Sullivan J (2013). Public knowledge of and attitudes toward genetics and genetic testing. Genet Test Mol Biomarkers.

[25] Ashida S, Goodman M, Pandya C, Koehly LM, Lachance C, Stafford J (2011). Age differences in genetic knowledge, health literacy and causal beliefs for health conditions. Public Health Genom.

[26] Hicks MA, Cline RJ, Trepanier MA (2014). Reaching future scientists, consumers, & citizens: what do secondary school textbooks say about Genomics & its Impact on Health?. Am Biol Teach.

[27] Lea DH, Kaphingst KA, Bowen D, Lipkus I, Hadley DW (2011). Communicating genetic and genomic information: health literacy and numeracy considerations. Public Health Genom.

[28] Lea DH, Skirton H, Read CY, Williams JK (2011). Implications for educating the next generation of nurses on genetics and genomics in the 21st century. J Nurs Scholarsh.

[29] American Nurses Association, International Society of Nurses in Genetics (2007). Genetics/ genomics nursing: scope and standards of practice.

[30] Godino L, Turchetti D, Skirton H (2013). Genetic counseling: a survey to explore knowledge and attitudes of Italian nurses and midwives. Nurs Health Sci.

[31] Godino L, Skirton H (2012). A systematic review of nurses’ knowledge of genetics. J Nurs Educ Pract.

[32] Tonkin E, Calzone K, Jenkins J, Lea D, Prows C (2011). Genomic education resources for nursing faculty. J Nurs Scholarsh.

