# Supplementary material for: Opportunities and challenges of integrating genetics education about human diversity into public health nurses’ responsibilities in Japan
Source: BMC Nurs. 2019 Dec 9;18:65. doi: 10.1186/s12912-019-0391-6 (PMC6902480; doi:10.1186/s12912-019-0391-6)
Supplement: Supplementary file 1 — Additional file 1. Interview guide developed for this study [file 12912_2019_391_MOESM1_ESM.docx]

Interview guide

Did you think it was difficult to answer the consultation?

Have you ever heard from your residents “Children have diabetes if diabetics are in the family”?

Did you think it was difficult to answer the consultation?

What skills are needed to solve the questions of genetic consultants?

When should the skills be developed as a public health nurse?

What did you answer at that time?

**You thought to be involved in genetic**: Did you think it was difficult to answer the consultation?

**You do not think as a genetic:** What are the reasons that you thought that there is no relation to the genetic?

Can you solve it?

Yes

No

Have you had any consultation or support related to genetic in the activities of public health nurses?

What kind of consultation?

What do you imagine about genetic counseling?

Why did you have that image?

Yes

No
